# Outcomes of patients with gallbladder cancer presenting with acute cholecystitis

**DOI:** 10.1007/s00423-024-03261-8

**Published:** 2024-02-23

**Authors:** Nunzio F Franco, Ngee-Soon Lau, Wai M Liu, Aadil Rahim, Mitali Fadia, Yu Jo Chua, Ankit Jain, Desmond Yip, Sivakumar Gananadha

**Affiliations:** 1 Department of Surgery, Canberra Health Services, Canberra, Australia; 2 School of Medicine and Psychology, Australian National University, Canberra, Australia; 3 Research School of Finance, Actuarial studies and Statistics, Australian National University, Canberra, Australia; 4 Department of Anatomical Pathology, Canberra Health Services, Canberra, Australia; 5 Department of Medical Oncology, Canberra Health Services, Canberra, Australia

**Keywords:** Gallbladder malignancy, Gallbladder cancer, Acute cholecystitis, Intraoperative spillage

## Abstract

**Abstract:**

The main purpose of this study is to explore the outcomes of patients found to have gallbladder cancer during investigation and diagnosis of acute cholecystitis. The incidence of primary gallbladder cancer co-existing in acute cholecystitis is not well defined in the literature, with anecdotal reports suggesting that they experience worse outcomes than patients with gallbladder cancer found incidentally.

**Methods:**

A retrospective review of all patients with gallbladder cancer managed at the Canberra Health Service between 1998 and May 2022 were identified and reviewed.

**Results:**

A total of 65 patients were diagnosed with primary gallbladder cancer during the study period with a mean age of 70.4 years (SD 11.4, range 59–81.8 years) and a female preponderance (74% versus 26%) with a ratio of 2.8. Twenty (31%) patients presented with acute calculus cholecystitis and were found to have a primary gallbladder cancer. This group of patients were older and predominantly female, but the difference was not statistically significant. The overall 5-year survival in the cohort was 20% (stage 1 63%, stage 2 23%, stage 3 16%, and stage 4 0%). There was no statistically significant difference in survival between those who presented with acute cholecystitis vs other presentations.

**Conclusions:**

A third of the patients with gallbladder cancer presented with acute cholecystitis. There was no statistically significant difference in survival in those with bile spillage during cholecystectomy as well those presenting with acute cholecystitis.

## Introduction

Gallbladder cancer represents a rare malignancy, accounting for only 0.6% of total cancer incidence worldwide and contributing to 0.9% of all cancer-related deaths [1]. It is the most prevalent cancer within the biliary tract [2]. The epidemiology of gallbladder cancer exhibits significant geographical variation, influenced by diverse environmental, genetic, and medical factors. Elevated incidence rates are documented in South Asia, East Asia, high-income Asia-Pacific nations, and Western Europe, while lower rates are observed in Sub-Saharan Africa and Australasia [3, 4]. The highest reported incidence rates occur in women from Chile (27/10,000), Delhi, India (21.5/100,000), and South Karachi, Pakistan (13.8/10,000), while the incidence decreases in high-income countries, such as the US and Australia [5] .

In Australia, gallbladder cancer maintains an incidence ranging from 2.8 to 3.7 per 100,000, with a relatively stable trend over the past three decades. Data sourced from the Australian cancer database delineate the variation in incidence across each state and territory over time [6]. Various other risk factors have been described such as gallstones [7], chronic infections (especially by Salmonella typhi species) [8], primary sclerosing cholangitis [9], obesity [10], and gallbladder polyps [11]. Despite the overall incidence appears on the rise, especially in younger individuals, the overall mortality rate is decreasing [4].

The diagnosis of gallbladder cancer can be made following investigation for undifferentiated abdominal pain, incidentally on imaging for a different pathology, or following cholecystectomy due to gallstone disease. The incidence of primary gallbladder cancer diagnosed during cholecystectomy for acute cholecystitis is not well defined in the literature. A recent study involving 218 patients reported that 37 patients had concomitant acute cholecystitis, indicating a propensity for these patients to experience a shorter overall survival, although statistical significance was not observed [12]. Anecdotally, these patients are still reported to have worse outcomes. This is attributed to the likelihood of malignancy seeding to the peritoneum or port sites due to laparoscopic manipulation, as well as the biological environment created by inflammation [13, 14]. This is believed to be the case particularly if intraoperative spillage occurs during the procedure, leading to peritoneal carcinomatosis [15]. The current study aims to look at the incidence and outcomes of patients with gallbladder cancer presenting with acute cholecystitis in an Australian tertiary hospital.

## Materials and methods

All patients diagnosed with gallbladder cancer between 1998 and May 2022 were identified from the hospital medical records, oncology database and the database of the ACT Pathology. Canberra Hospital is a tertiary referral hospital serving the population of the Australian Capital Territory and the surrounding southern New South Wales. This study was Approved by ACT Health Human Research Ethics Committee (2022.LRE.00157).

Clinical data extracted were preoperative patient characteristics, patient presentation, preoperative imaging, and histological data. Operative data included the type of surgery including occurrence of intraoperative rupture of the gallbladder or bile spillage during surgical intervention and complications. Acute cholecystitis was defined using the Tokyo guidelines 2018 [16]. Postoperative data included adjuvant therapy and patient outcomes and mortality at a cut-off date of 1st of May 2022. Gallbladder cancer staging was performed in accordance with the American Joint Committee on Cancer (AJCC) 8th edition [17]. Patients were discussed at multi-disciplinary meetings and management according to the NCCN Guidelines current at the time of presentation [18]. Patients underwent re-resection depending on the histology and the ability to achieve a curative negative margin on restaging investigations.

Continuous data were expressed as means and standard deviations (or medians and interquartile (IQR) ranges). Differences in continuous variables were assessed using Student’s *t*-test or Mann-Whitney *U* test. Pearson’s Chi square test (or Fisher’s exact test) was used to compare proportions. Survival was determined by Kaplan Meier method and differences in survival were analyzed by means of the log-rank test Wilcoxon-Breslow-Gehan test. Univariate analyses were performed using cox proportional hazard regression. Incidence was assessed by considering the mid-period population of the ACT, more specifically in 2010. A significance level of 0.05 was used for all analyses. All data management and statistical analyses were executed in STATA 16 statistical software (StataCorp LP, College Station, TX).

## Results

During the study period, a total of 65 patients were diagnosed with primary gallbladder cancer. The mean age of the patients was 70.4 years (SD 11.4, range 59–81.8 years). The demographics of the study population is shown in Table 1*.* Primary gallbladder cancer was more predominant in females compared to male (74% versus 26%) with a ratio of 2.8. The majority of the patients identified as Australian (66%) with the rest comprised of various European nationalities, and one case occurring in an Indian patient. The average population of the ACT during the study period of 1998–2022 was 369,369.21 [19], while in 2010, the population of the ACT was 358,222; the incidence of primary gallbladder cancer was therefore 0.75 per 100,000.

**Table 1:** Demographic data of the cohort

	Overall	
	*N*/mean	%/SD
Total	65	100%
Age [mean, SD]	70.4	11.4%
Gender		
Female	48	74%
Male	17	26%
Nationality		
Australian	43	66%
Others	22	34%
AJCC staging (for primary)		
I	11	17%
IIA	5	8%
IIB	5	8%
IIIA	12	18%
IIIB	11	17%
IVA	1	2%
IVB	20	31%
Management		
*Intent*		
Curative	33	51%
Palliative	32	49%
*Surgery*		
Surgery only	25	38%
Surgery and adj. chemo.	18	28%
Surgery and adj. radio.	4	6%
*Non-operative*		
Chemotherapy only	6	9%
Radiotherapy only	0	0%
No treatment offered	11	17%
Presentation	20	31%
Cholecystitis		
Other	45	69%
Intraoperative spillage	18	38%

^§^*t*-test for *Age*. For other categorical variables, Pearson χ^2^ is used

### Presentation

In the study period, 20 patients presented with acute calculus cholecystitis (31%) and were found to have a primary gallbladder cancer. This group of patients were older and predominantly female, but the difference was not statistically significant (Table 2). There was no difference in the stage of presentation between the group diagnosed with gallbladder cancer during cholecystectomy and the other group. Other presentations included asymptomatic incidental findings following imaging for other indications and imaging for non-specific abdominal pain (69%).

**Table 2:** Demographics by presentations

	Presentation				*p*-value^§^
	Cholecystitis		Other presentation		
	*N*/mean	%/SD	*N*/mean	%/SD	
Age [mean, SD]	71.67	12.35	69.82	11.01	0.55
Gender					0.17
Female	17	85%	31	69%	
Male	3	15%	14	31%	
Nationality					0.12
Australian	16	80%	27	60%	
Others	4	20%	18	40%	
AJCC staging (for primary)					0.79
I	3	15%	8	18%	
IIA	2	10%	3	7%	
IIB	2	10%	3	7%	
IIIA	4	20%	8	18%	
IIIB	5	25%	6	13%	
IVA	0	0%	1	2%	
IVB	4	20%	16	36%	
Management					0.33
*Surgery*					
Surgery only	9	45%	16	36%	
Surgery and adj. chemo.	6	30%	12	27%	
Surgery and adj. radio.	2	10%	2	4%	
*Non-operative*					
Chemotherapy only	0	0%	6	13%	
Radiotherapy only	0	0%	0	0%	
No treatment offered	3	15%	9	20%	
Intraoperative spillage	13	76%	5	17%	

^§^*t*-test for *Age*. For other categorical variables, Pearson χ^2^ is used

### Staging

The majority of the patients were stage III or IV (68%), and the rest were stage I or II (32%). The stage distribution of the group was 11 patients presented with stage I (17%), 10 patients presented with stage II, 5 stage IIA (8%) and 5 stage IIB (8%), 23 were found to have stage III, with 12 patients (18%) stage IIIA, and 11 patients (17%) with stage IIIB. Finally, 21 patients were found to have stage 4 gallbladder cancer, with 1 patient with stage IVA (2%) and 20 patients with stage IVB (31%). In the cohort, 32 patients were offered palliative intent management, including no active treatment or palliative chemotherapy or radiotherapy, while 33 patients were offered treatment with curative intent.

### Management

Management for gallbladder cancer was mostly surgical, with 47 patients undergoing a cholecystectomy (72%). Of these, 21 patients had a radical cholecystectomy with portal lymphadenectomy and liver bed resection, and the rest had a simple cholecystectomy (26 patients). Majority of patients presenting with acute cholecystitis only had simple cholecystectomy (13 patients) with 4 patients having radical cholecystectomy. Thirty-eight percent of patients underwent surgical treatment only, 28% received surgical treatment and adjuvant chemotherapy, and 6% received chemotherapy and adjuvant radiotherapy. Of the 47 patients who underwent surgical treatment, intraoperative spillage occurred in 18 cases (38%). Twenty-six percent of patients did not undergo surgical management, with 9% receiving chemotherapy only, while 17% underwent no treatment.

### Survival

**Fig. 1:**
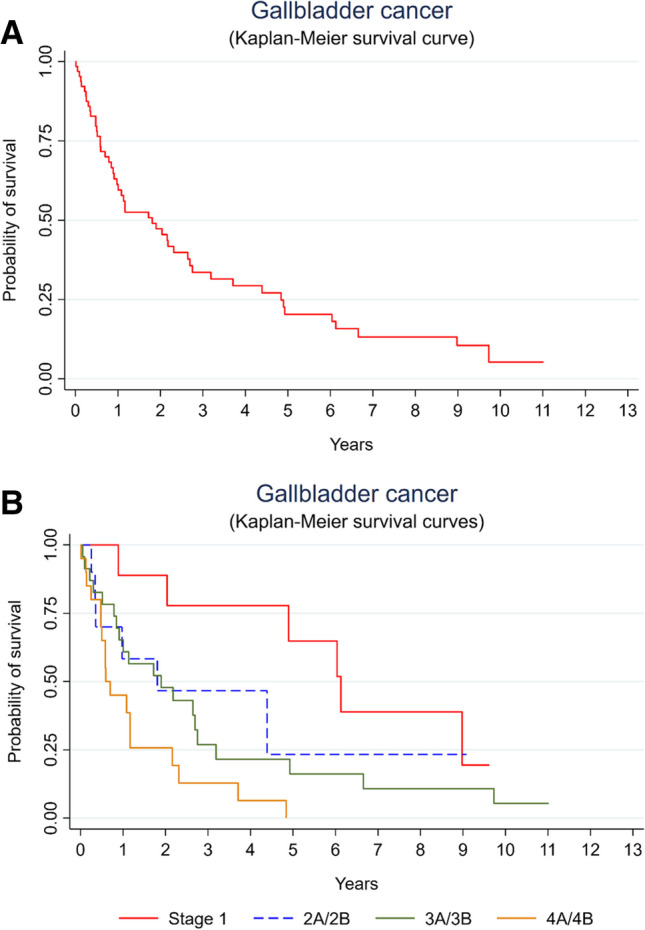
Kaplan-Meier survival curve for gallbladder cancer according to AJCC8 staging

The 5-year survival in the overall cohort was at 20%, as seen in Fig. 1A. When adjusted by stage, survival curves are significantly different across staging with a *p* value of 0.002 for log rank test and a value of 0.006 for Wilcoxon-Breslow-Gehan test (Fig. 1B). The 5-year survival for stage 1 gallbladder cancer was of 63%, for stage 2A and 2B at 23%, and for stage 3A and 3B at 16%, and finally no participant was alive at 5 years with stage 4A and 4B gallbladder cancer.

**Fig. 2:**
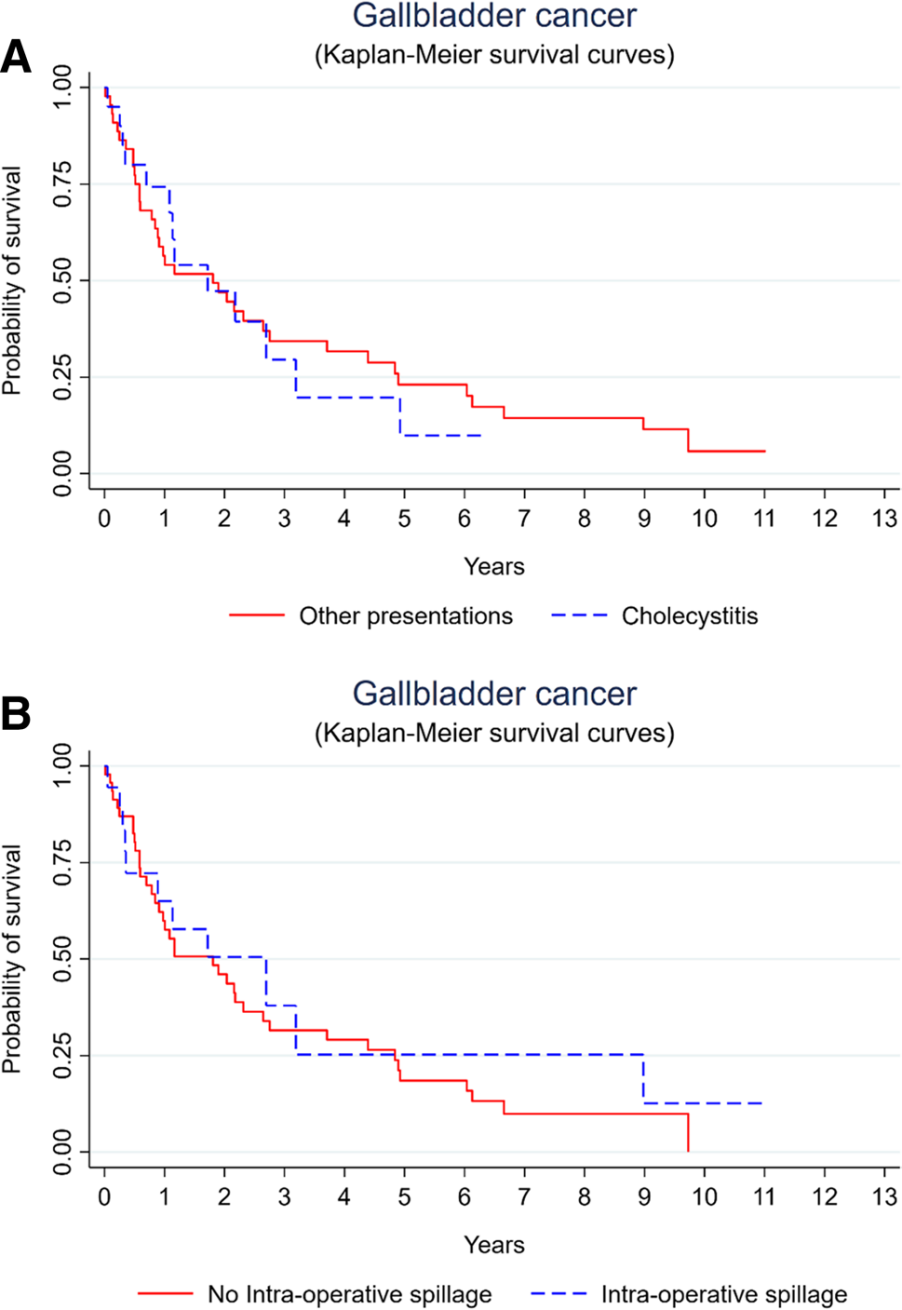
Kaplan-Meier survival curve for gallbladder cancer with acute cholecystitis and for intraoperative spillage

In our cohort, there is no statistically significant difference for patients who presented with acute cholecystitis and were found to have gallbladder cancer when compared to those with other presentations, such as abdominal pain or incidental findings, as seen in the survival curve in Fig. 2A. The *p* value for log rank test was found to be 0.88, while the Wilcoxon-Breslow-Gehan test was found to be 0.81

For the patient who underwent surgical treatment with initial cholecystectomy with or without completion radical cholecystectomy, there was no difference in the survival curve for those who were reported to have had intraoperative spillage, compared to those who had no spillage (Fig. 2B). The *p* value for log rank test was found to be 0.52, while the Wilcoxon-Breslow-Gehan test was found to be 0.79.

## Discussion

This study describes the demographic, presentation, management, and survival of patients diagnosed with gallbladder cancer in the Australian Capital Territory (ACT). Gallbladder cancer is a rare malignancy in the Australian population and carries a poor prognosis. When considering the mid-period population of the ACT, the incidence rate is calculated at 0.75 per 100.000 people. The median age of diagnosis was 70.4 years, with 74% of the patients being female. These findings are consistent with the incidence and age at diagnosis reported in western countries such as the USA and Canada. In fact, the incidence rate in these geographical areas is reported to be less than 2 per 100,000 [20], while the median age of gallbladder cancer diagnosis in Australia is reportedly between 67 and 73 years old [6].

Gallbladder cancer affects women disproportionately, as noted by previous studies [21, 22]. It is not well described why female patients have a higher rate of gallbladder cancer, but it is possibly due to risk factors such as gallstones, early menarche, late menopause, early first childbirth, and likely estrogen and progesterone receptors participating in the pathogenesis of GBC [22]. In our cohort, we found that 74% of patients were female, a number consistent with previous reports [21, 22].

Most of our cohort was found to have local spread or metastatic disease at presentation, with 68% of patients presenting with stage III or IV. Thirty-one percent of the cohort was found with distant metastasis at presentation, consistent with stage IVB. This high proportion is expected as gallbladder cancer is mainly asymptomatic until late stages, where local invasion or distant metastatic disease can cause symptoms [23]. On the contrary, early gallbladder cancer is often found incidentally after cholecystectomy or imaging performed for different indications, with better outcomes for these patients [15].

In the Australian Capital Territory, an estimated 659 laparoscopic cholecystectomies were completed in 2014–2015 per 100,000 people, the lowest number of all the Australian states and territories. Although the incidence of incidental finding of gallbladder cancer after cholecystectomy is only found to be approximately 0.7–0.8% in western countries [15, 24], it is expected that with a growing number of elective and emergency cholecystectomies, the number of late-stage presentation of gallbladder cancer would decrease. In Australia, the total number of cholecystectomies has increased from 46,332 in 2000–2001 to 60,470 in 2020–2021. This is a 30.5% increase for this procedure according to the Australian Institute of Health and Welfare [25]. This hypothesis is supported by the link between cholecystectomy and gallbladder cancer which has been suggested before, when a decrease in the rate of this procedure was thought to be responsible for the rise in the incidence of gallbladder cancer in the 1980s and 1990s in South America [26, 27]. Future research is needed to assess if the rising numbers of cholecystectomies will result in a lower rate of late-stage presentations for gallbladder cancer.

We found an overall survival rate for all patients of 20% at 5 years, consistent with a high mortality malignancy. This is similar to recent reports of overall survival in western reports [28, 29]. We found statistically significant differences in survival between stages, with 63% of patients with stage 1 disease alive at 5 years, while no patient with stage 4 disease survived at 5 years. These results reflect and align with validation of the recently updated staging system, which has found similar differences in survival according to stages [17]. The AJCC 8^th^ edition has recently been updated from the previous 7^th^ edition, which has now divided stage II into IIA and IIB (Image 1). The main update reflects evidence that a malignancy located on the hepatic side (IIB) has a worse prognosis than one located on the serosal side (IIA), possibly due to a higher neural invasion and lymph node involvement [17]. This difference was not noted in the validation study. Our cohort was too small to attempt to find a difference in survival between stage IIA and IIB, which requires a higher number of subjects, a limitation of this study.

Preoperative inflammation of the gallbladder and a high neutrophil-lymphocyte ratio are considered negative prognostic factors in gallbladder cancer [14, 30]. These findings contribute to the idea that patients presenting with acute cholecystitis who undergo cholecystectomy and are found to have gallbladder cancer intraoperatively or on histopathology have a worse prognosis than those found incidentally. The explanation for this is not described, but it is suggested that it could be due to a higher likelihood of angiolymphatic or perineural invasion [14].

In our current cohort, 31% of patients underwent cholecystectomy for acute cholecystitis and were incidentally found to have gallbladder cancer on routine histopathological examination. We showed no statistically significant difference in survival between for those presenting with acute cholecystitis. Similarly, Kihara et al. recently showed a tendency for these patients to have worse overall survival, although this was not statistically significant [12]. Despite our results align, given our limited numbers, our results must be interpreted with caution.

Intraoperative spillage during laparoscopic cholecystectomy is thought to confer a poorer prognosis for gallbladder cancer, secondary to possible spillage of malignant cells within the peritoneal cavity and seeding of the peritoneum leading to peritoneal carcinomatosis. Multiple reports have shown that patients who develop this intraoperative complication have a worse outcome, although the numbers available are often low [31, 32]. In our cohort, intraoperative spillage occurred in 38% of those undergoing surgical resection. These patients had no statistically significant survival difference compared to those without this complication.

We acknowledge several limitations of this paper. The study population is small, a statistically limiting factor. Given the low incidence of this malignancy, it is not possible to achieve a higher number of study subjects. The study does not report on the histological subtype of gallbladder cancer, an important prognostic factor. The incidence rate was calculated based on the population of the ACT. An estimation of the population with the catchment area is likely to be inexact given a wide draining area and give a false incidence rate. Laboratory findings were not included in the data collection and hence could not be included in the analysis. Patients with gallbladder cancer presenting with acute cholecystitis is not an uncommon presentation in a Western population with high incidence of cholelithiasis and low incidence of gallbladder cancer. There seems to be no significant difference in outcomes; however, the results need to be interpreted with caution due to the small numbers.

## Conclusion

Gallbladder cancer is a rare cancer, often found incidentally. Patients who present with acute cholecystitis or experience intraoperative spillage during surgical management are believed to have a worse prognosis. This small retrospective study shows no difference in survival between patients who are incidentally found to have gallbladder cancer versus patients who present with acute cholecystitis, and for those experiencing intraoperative spillage. The small numbers of the study poses obvious limitations to this interpretation, with future research necessary to corroborate our findings.

## Data Availability

Data is available upon request from the corresponding author.
